# Identification of the Major Degradation Pathways of Selumetinib

**DOI:** 10.3390/pharmaceutics14122651

**Published:** 2022-11-30

**Authors:** Tahar Sif eddine Bouchema, Maxime Annereau, Victoire Vieillard, Raphael Boquet, Gisele Abreu Coelho, Florence Castelli, Audrey Solgadi, Muriel Paul, Najet Yagoubi, Philippe-Henri Secretan, Bernard Do

**Affiliations:** 1 Matériaux et Santé, Université Paris-Saclay, 91400 Orsay, France; 2 Clinical Pharmacy Department, Gustave Roussy Cancer Campus, 114 Rue Edouard Vaillant, 94800 Villejuif, France; 3 Department of Pharmacy, Henri Mondor Hospital, AP-HP, 94000 Créteil, France; 4 Département Médicaments et Technologies pour la Santé (DMTS), MetaboHUB, CEA, INRAE, Université Paris-Saclay, 91191 Gif-sur-Yvette, France; 5 Ingénierie et Plateformes au Service de l’Innovation Thérapeutique, Inserm, CNRS, Université Paris-Saclay, 92296 Châtenay-Malabry, France; 6 EpidermE, Université Paris Est Creteil, 94010 Creteil, France

**Keywords:** pre-formulation studies, intrinsic stability, degradation pathways, oxidation, photooxidation, protein kinase inhibitor, ESI-MS fragmentation pathways

## Abstract

Selumetinib is administered orally in capsule form and is indicated for the treatment of neurofibromatosis. To facilitate dosage adjustments, liquid preparations, such as solutions or suspensions, are to be developed. This led, first, to determine the stability profile of soluble or dispersed selumetinib and, secondly, to look for ways to stabilize the active substance. The degradation kinetics of selumetinib as a function of stress conditions were determined and compared. The degradation products were detected and identified by LC-HRMS^n^. In solution, selumetinib is sensitive to oxidation and degrades by photooxidation. In both cases, the side chain represented by the oxoamide group is concerned, leading to the formation of an amide derivative for the first case and an ester derivative for the second. The identification of such degradation mechanisms allowed us to study, in a targeted way, processes aiming at stabilizing the active molecule.

## 1. Introduction

Drugs that act through protein kinase inhibition are indicated for the treatment of solid tumors, leukemias, and inflammatory diseases, such as rheumatoid arthritis [1,2]. Depending on their mechanism of action, the molecules target either protein serine/threonine kinases, dual-specific protein kinases involved in the MAPK signaling pathway (MEK1 and MEK2), non-receptor protein tyrosine kinases, or receptor protein tyrosine kinases [3].

Selumetinib (Koselugo^®^, (6-(4-bromo-2-chloroanilino)-7-fluoro-N-(2-hydroxyethoxy)-3-methylbenzimidazole-5-carboxamide) belongs to the class of products that inhibit MEK1 and MEK2 proteins [3,4], which are hyperactivated in NF-1 [5]. The latter has been approved for the treatment of neurofibromatosis type I (NF-1) [6], a genetic disease of the nervous system causing tumor growth on nerves [7] and inducing the development of localized skin neurofibromas [8]. However, like other MEK1/2 inhibitors, this drug is only available in solid dosage form [3]. Yet, there is increasing evidence of parallel development of other forms in the sense that topical administration, for example, appears to allow regression of cutaneous squamous cell carcinoma [9,10], hand eczema [11], and inhibit corneal neovascularization [12], while reducing the risk of systemic side effects. For this, it is, above all, crucial for the formulator to have access to the behavior of selumetinib in terms of stability, at least in solution or in suspension at first.

After a thorough review of the literature, it appears that there is very little published work on the stability of selumetinib, with the understanding that this work was performed primarily in the context of developing stability-indicating methods for the determination of selumetinib in capsules, where it is important to obtain degradation products allowing the evaluation of the method’s selectivity [13]. Other sources, such as the U.S., European, and Japanese pharmacopeias, where information on related substances is often found, do not include a monograph on selumetinib.

Rapid access to such knowledge is often provided by studying the behavior of the drug substance under stress conditions and characterizing the products resulting from the degradation of the parent molecule [14,15]. Based on our preliminary studies, selumetinib degrades very readily under oxidative and simulated daylight conditions.

As a result, to go further and understand the degradation mechanisms involved, we proceeded to the structural elucidation of the main degradation products by liquid chromatography coupled with high-resolution mass spectrometry (LC-HRMS), a recognized technique for the structural elucidation of degradation products [16,17]. This research has allowed the characterization of the main processes involved in the degradation of selumetinib. Based on these results, potential measures to overcome the instability of selumetinib in solution or suspension toward photodegradation and oxidation could be explored.

## 2. Materials and Methods

### 2.1. Reagents

Selumetinib (purity > 99%) was purchased from MedKoo Biosciences (Morrisville, NC, USA). Analytical grade acetonitrile came from Sigma-Aldrich (St Quentin-Fallavier, France). Ultrapure water was produced by the Q-Pod Milli-Q system (Millipore, Molsheim, France). Hydrogen peroxide (H_2_O_2_) 30% *v*/*v* was supplied by Carlo Erba SDS (Val de Reuil, France) whereas analytical grade hydrochloric acid, sodium hydroxide, and alpha-tocopherol acetate were obtained from Sigma-Aldrich (St Quentin-Fallavier, France).

### 2.2. Preparation of Solutions

A stock solution was prepared by dissolving selumetinib in pure acetonitrile (final concentration = 500 µg·mL^−1^). This solution was then diluted and exposed to simulated light and oxidative conditions.

For photodegradation studies, three solutions were prepared by diluting the stock solution in water to reach a final concentration of 250 µg·mL^−1^ of selumetinib. However, before adjusting to the final volume, hydrochloride acid and/or sodium hydroxide were added to the mixture to adjust their pH to 3, 6, and 9. Thereafter, these solutions were exposed to simulated light using a Q-SUN Xe-1 xenon test chamber (Q-Lab Corporation, Saarbrücken, Germany) operating in window mode and conforming to ICH Q1B recommendations.

To study the impact of oxidative stress, two solutions were prepared by diluting the stock solution in water and adding H_2_O_2_ to reach a final concentration of 250 µg·mL^−1^ of selumetinib and a final concentration of H_2_O_2_ of 3% and 0.3%. For kinetic studies, the oxidation of Selumetinib was studied by exposing the solution containing 3% of H_2_O_2_ at three different temperatures: 40, 50, and 60 °C. The impact of tocopherol was studied by adding alpha-tocopherol acetate to reach a final concentration of 500 µg·mL^−1^.

The impact of hydrolytic stress was studied by preparing two solutions using the stock solution, water, hydrochloric acid, and sodium hydroxide to obtain a final concentration of 250 µg·mL^−1^ of selumetinib with pH set at 1 and 13. These solutions were stored at 40 °C and repeatedly analyzed for fourteen days.

### 2.3. Instrumental

This section concerns the experimental conditions applied to the determination of selumetinib, the detection of degradation products (HPLC-UV), and the study of their structure (LC-MS). The chromatographic separation, upstream of the double UV and MS detection, was performed using a Dionex UltiMate 3000 system (Les Ulis, France), which was controlled and acquired by the Chromeleon^®^ software, version 6.80 SR11 (Dionex, Les Ulis, France). The stationary phase was a Phenomenex C18 column (250 nm × 4.6 nm; 5 µm) placed in an oven set at 30 °C. The mobile phase consisted of a gradient combining 0.1% (*v*/*v*) formic acid in ultrapure water (solvent A) and 0.1% (*v*/*v*) formic acid in acetonitrile (solvent B) where mixing was performed according to the following program: 0–2 min: 95% A; 2–30 min: 95 → 0% A; 30–35 min: 95% A. For UV detection at 220 nm, the flow rate at the column outlet remained unchanged, i.e., 1 mL min^−1^. However, the flow going into the mass spectrometer was reduced to 0.3 mL min^−1^ by using a 1/3 T fractionator positioned at the outlet of the UV detector.

Orbitrap™ Q Exactive™ Plus (Thermo Fisher Scientific, Waltham, MA, USA) was used for mass spectrometric analysis of the eluted compounds, the tuning data of which are shown in the supplemental material (Table S1). High-resolution mode analyses were performed over the mass range of 80 to 1200 amu. The total width at the half height selected (measured at fifty percent of the maximum peak height) was 140,000 and 70,000 for HR-MS and HR-MS², respectively. The MS data were processed using Xcalibur^®^ software (version 2.2 SP 1.48).

## 3. Results and Discussion

### 3.1. General Susceptibility of Selumetinib to Various Stress Conditions

**Figure 1:**
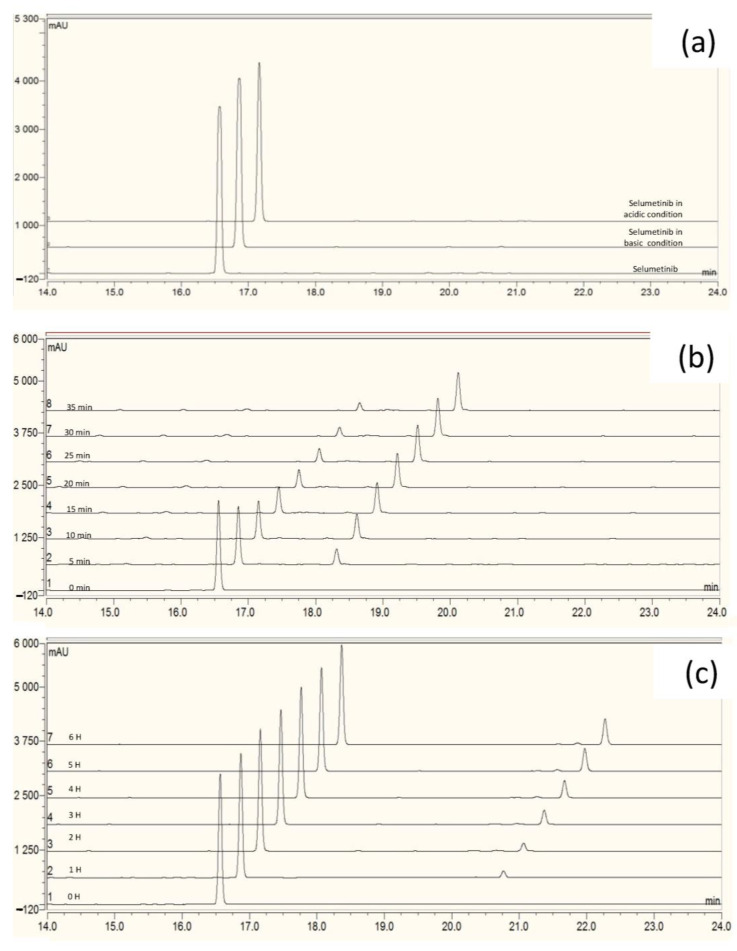
Inset (**a**): chromatograms of freshly prepared selumetinib solution (black plot), selumetinib solution exposed to acidic conditions for one week (blue plot) and selumetinib solution exposed to basic conditions for one week. Inset (**b**): chromatograms of selumetinib solution exposed to light irradiation obtained after analysis of samples taken every 5 min for 35 min. Inset (**c**): chromatograms of selumetinib solution exposed to oxidative stress (3% H_2_O_2_, 40 °C) obtained after the analysis of samples taken every hour for at least 5 h.

The drug was exposed to various stress conditions, and its concentration was monitored as a function of time. The same separation conditions were applied for UV and mass detection, thus allowing complementary control of peak purity. There were no co-eluted products. Mass balance was consistently achieved for all analyses. After 14 days of exposure to hydrolytic stress, no loss of selumetinib and, consequently, no appearance of degradation products was observed (Figure 1a). This resistance to hydrolytic conditions is not consistent with data from forced degradation in the context of stability indicating method development [13]. This is because our pH conditions were deliberately made less harsh to be closer to pH values compatible with formulation and administration.

In contrast, we found that photolytic stress and oxidative stress gave rise to the formation of two main degradation products, named “DP1” (Figure 1b) and “DP2” (Figure 1c), respectively.

In the absence of degradation products other than DP1 and DP2, these were subsequently used as critical tracers, or quality attributes to monitor the stability of selumetinib.

The oxidative degradation kinetics of selumetinib was also studied in the presence of 3% H_2_O_2_ and at three different temperatures (40, 50, and 60 °C) in triplicate. The plot of the percentage of the remaining active substance concentration expressed as a logarithmic value versus time (minute) is a linear relationship, which corresponds to pseudo-first-order kinetics. The apparent order degradation rate constant values were respectively 0.0007, 0.0019, and 0.0035 min^−1^ at 40, 50 and 60 °C, respectively. The activation energy value obtained for the oxidative degradation of selumetinib is 0.1937 kJ.mol^−1^.

**Figure 2:**
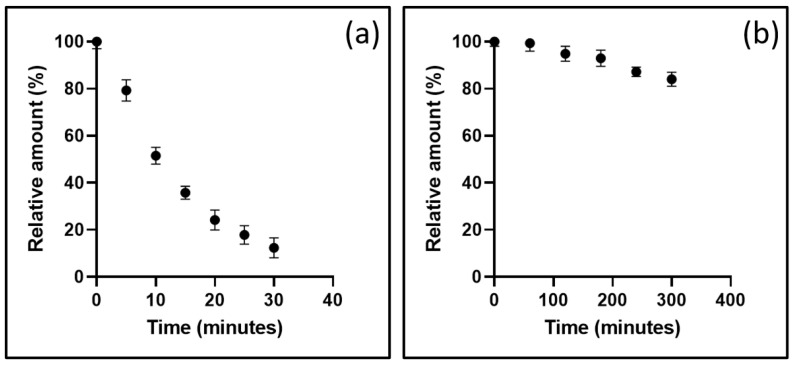
Evolution of selumetinib (relative amount) in solution under stress conditions (exposition to simulated light at 25 °C) (inset (**a**)) and in the presence of H_2_O_2_ (3%) at 40 °C (inset (**b**)).

In summary, these results highlight the high sensitivity of selumetinib in solution to light irradiation (Figure 2a) and oxidative conditions (Figure 2b).

### 3.2. Structural Characterization of DP1 and DP2

**Scheme 1:**
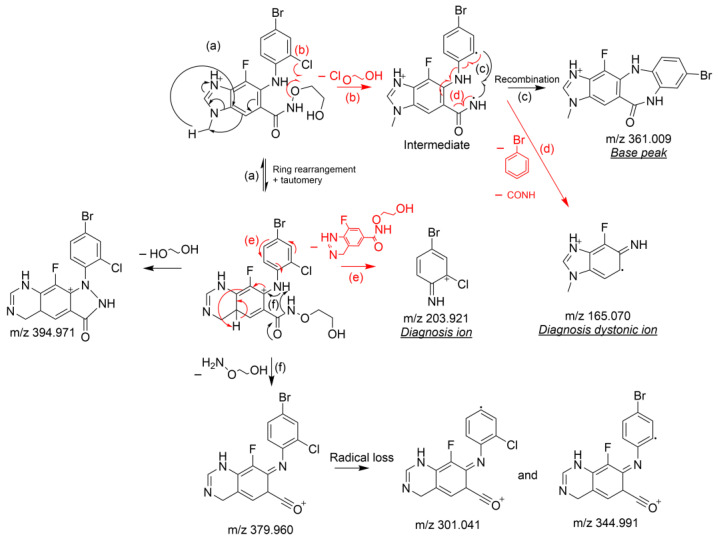
Fragmentation pattern of protonated selumetinib.

**Scheme 2:**
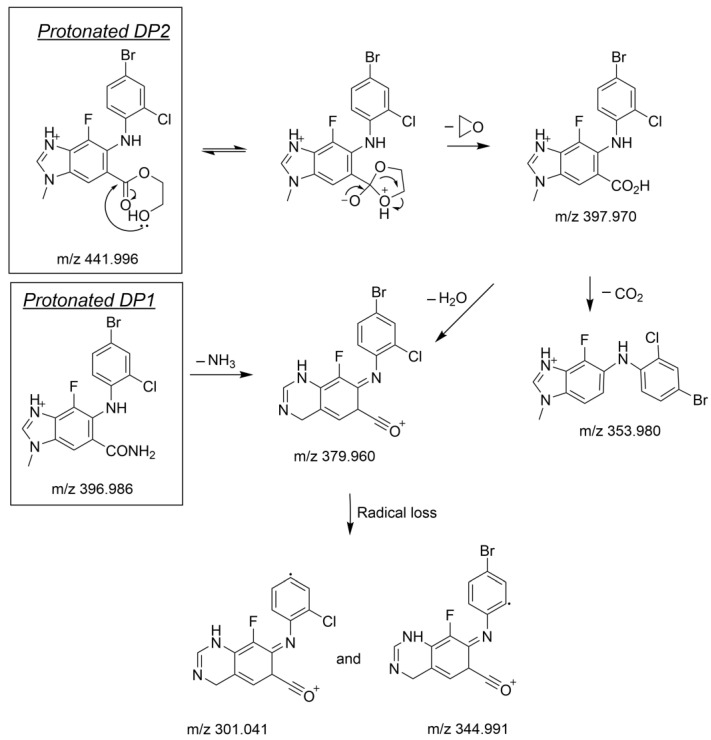
Fragmentation pattern of protonated DP1 and DP2.

The structural elucidation of DP1 and DP2 is a deductive approach comparing neutral and/or radical losses to those characterizing selumetinib under the same analytical conditions. A comprehensive study of selumetinib’s fragmentation pattern was carried out, being a key part of the degradation products identification process [18]. The product ions’ structures of selumetinib (Scheme 1) and of its degradation products (Scheme 2) were systematically confirmed through the elemental composition determination based upon accurate mass measurement (Figures S1–S5). The exact mass of each proposed structure in the fragmentation patterns (Scheme 1 and Scheme 2) matches that of an accurate mass detected (Figures S1–S5) with a difference of 1 mDa at most.

#### 3.2.1. Specific Fragmentation Pattern of Selumetinib

The characteristic fragmentation pattern (Scheme 1) of selumetinib was proposed after an in-depth study of its mass spectrum provided in the supplementary information (Figure S1). The structures and mechanisms of the formation of daughter ions and neutral loss of selumetinib that contributed to the structural elucidation of the DPs are detailed hereafter.

The LC-HRMS^2^ mass spectrum of selumetinib’s protonated ion (C_17_H_16_BrClFN_4_O_3_^+^; degree of unsaturation, DU = 10.5) presents seven intense daughter ions whose elemental formulas and tentatively identified structures are presented in Figure S1. It should be noted that the mass spectra of protonated DP1 and DP2 (Figures S3 and S5) also exhibit four of these daughter ions, i.e., *m/z* 379.960, 344.991, 301.041, and 203.921, and this finding allowed us to comparatively deduce the common parts of the three molecules, i.e., the parts of selumetinib that were unchanged after its oxidation or photolysis. The analysis of the ions only detected for DP1 or DP2, i.e., specific to the latter, allowed to determine their elemental compositions, to deduce the structure of the fragments concerned, and then to reconstruct in a plausible way the chemical structures of DP1 and DP2, as presented below.

##### Characterization of Selumitinib Daughter Ions Not Detected for DP1 and DP2 (*m/z* 394.971, 361.010 and 165.070)

These three daughter ions were characteristic of changes occurring on the aliphatic side chain of the molecule (e.g., N-(2-hydroxyethoxy)formamide).

Based on its accurate mass (361.010), the base peak ion’s formula corresponds to C_15_H_11_BrFN_4_O^+^ (DU = 11.5). However, as chlorine is bound to an aromatic group, its departure by heterolytic cleavage is difficult to envisage. On the other hand, it seemed that its radical loss concomitantly with that of the hydroxyl-ethyl group is plausible (Scheme 1, path (b)), thus leading to an intense daughter ion represented by the base peak. This means that the absence of this base peak in the DPs’ mass spectra is directly linked to the modification of the aliphatic side chain (see Section 3.2.2).

The mechanism of formation of the base peak was confirmed by the intense presence of a dystonic ion (Scheme 1, path (d)), whose accurate mass (*m/z* 165.070) is consistent with formula C_8_H_8_FN_3_^•+^. Indeed, this ion can easily be formed from the same intermediate depicted for the base peak ion (Scheme 1, path (b)) by a concomitant loss of isocyanic acid and 2-bromocyclohexa-1,3-dien-5-yne (Scheme 1, path (d)).

The ion characterized by *m/z* 394.971 corresponds to a neutral loss of ethane 1,2 diol. The absence of this neutral loss in the mass spectra of DP1 and DP2 again confirms that a change has occurred for both on the aliphatic portion of the molecule (e.g., N-(2-hydroxyethoxy)formamide).

##### Characterization of Selumetinib Daughter Ions Common with DP1 and DP2 (*m/z* 379.960, 344.991, 301.041, and 203.921)

The ion represented by *m/z* 203.921 is specific to the 4-bromo-2-chloroaniline ring. Its mechanism of formation is proposed in Scheme 1 (paths (a) and (e)).

The ion at *m/z* 379.960 is consistent with the molecular formula C_15_H_9_BrClFN_3_O^+^ (UF = 11.5). It results from the loss of 2-(aminooxy)ethan-1-ol creating an additional unsaturation forming an oxonium ion (Scheme 1, paths (a) and (f)). The presence of this ion and the other ions derived from it, by loss of a bromide radical (C_15_H_9_ClFN_3_O^•+^, 301.041) or a chloride radical (C_15_H_9_BrFN_3_O^•+^, 344.991) for DP1 and DP2, are unambiguously synonymous with the fact that they, with the exception of the aliphatic side-chain present on selumetinib, have retained the other parts of the selumetinib structure, confirming the information presented in the previous paragraph.

#### 3.2.2. Structural Elucidation of the Main Degradation Products of Selumetinib

The structural elucidation of DP1 and DP2 was based on the comparison of neutral and/or radical losses to those found for selumetinib. Scheme 2 depicts the fragmentation patterns supporting the structure of DP1 and of DP2. A detailed explanation of these fragmentation patterns and the supporting spectral data are gathered in the supplementary information.

### 3.3. Main Degradation Pathways of Selumetinib

**Scheme 3:**
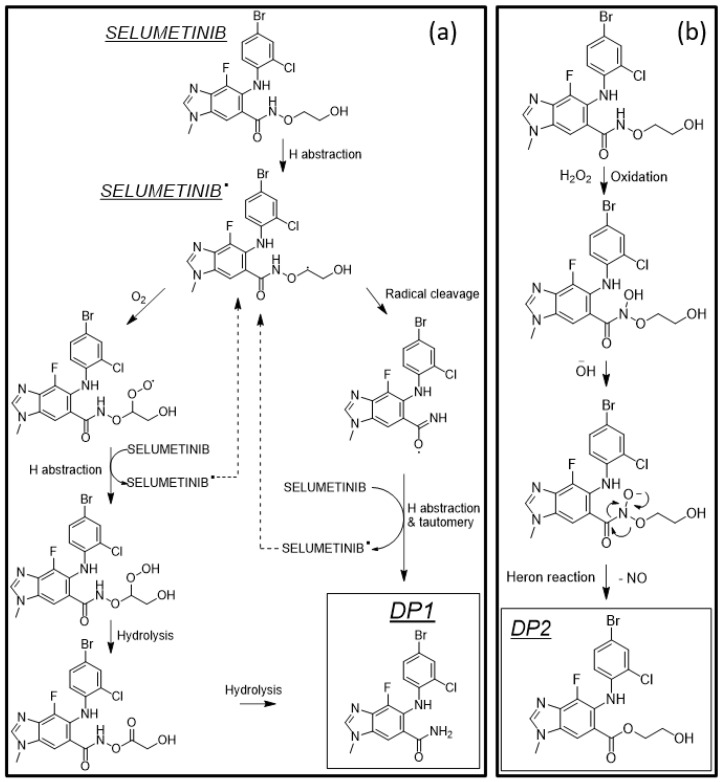
Degradation pathway of DP1 (inset (**a**)) and DP2 (inset (**b**)).

The main degradation pathways leading to the respective formation of DP1 and DP2 are depicted in Scheme 3.

Due to light exposition, h-abstraction occurred at the α carbon of the N-ethoxy function (Scheme 3a). Thereafter, radical selumetinib can easily undergo a direct cleavage initiated by the radical and react with water, for instance, to form DP1 (Scheme 3a). Another possibility involves dioxygen, as depicted in Scheme 3a. Noteworthy, in both cases, once the reaction has been initialized, it can be propagated (Scheme 3a), which is in line with the very fast degradation rate observed for selumetinib upon simulated light (Figure 2a).

In the presence of hydrogen peroxide, the nitrogen atom of the N-ethoxy is prone to forming an unstable hydroxylamine intermediate amenable to rearranging the yield into the very stable degradation product DP2 (Scheme 3b) by a heron reaction [19].

Although other explanations are possible, being able to propose mechanisms of degradation forming DP1 and DP2 under specific stress conditions participates in confirming the structures put forward for the degradation products.

### 3.4. Investigation of Measures to Reduce the Degradation of Selumetinib

**Figure 3:**
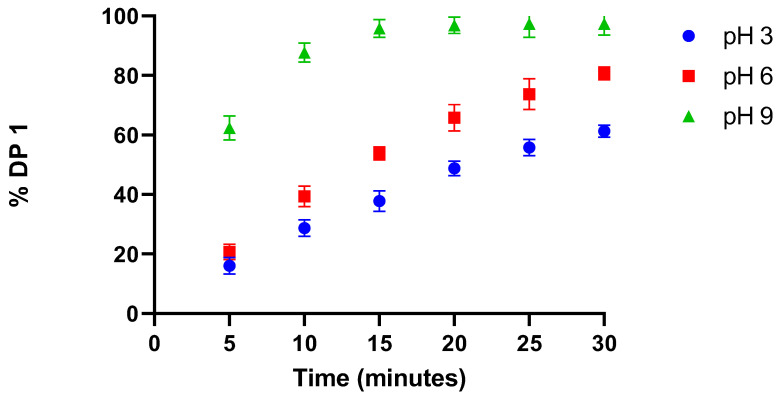
Percentage of degradation product formed under the simulated light condition (DP1) as a function of time and pH condition.

The influence of pH on selumetinib photodegradation was evaluated by following the variation of DP1 signal intensity as a function of exposure time and pH (Figure 3).

**Figure 4:**
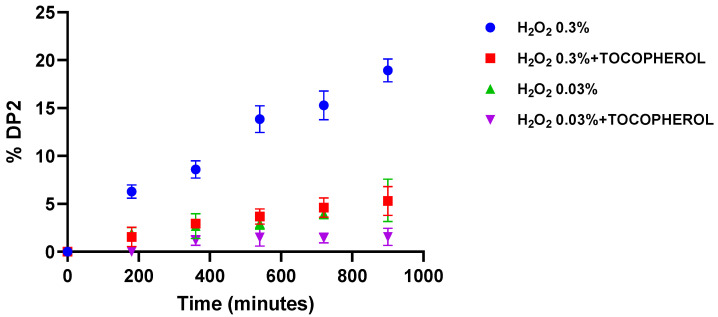
Percentage of degradation product formed under oxidative stress (DP2) as a function of time and in the presence of tocopherol.

The impact of a radical scavenger on oxidation, on the other hand, was evaluated by following the relative area of DP2 as a function of the exposure time (Figure 4).

Based on these results, the photodegradation of selumetinib is less intense in acidic pHs since the chromatographic profiles do not differ qualitatively as a function of pH; that is, we did not detect a change in the degradation pathways. The pH effect may be explained by the change in absorbance between 280 and 400 nm observed as a function of pH (Figure S6). Indeed, using the equation provided in OECD Guideline 316, the direct photolysis rate constant of selumetinib under natural solar light was determined to be 22,651 and 10,231 day^−1^ at pH = 3 and pH = 9, respectively.

Finally, the potential propensity of tocopherol, a radical scavenging compound [20], to reduce the degradation by oxidation was tested. The addition of this scavenger may clearly help to reduce the degradation of selumetinib in the presence of oxidizing agents (Figure 4).

Based on all these results, if a semi-solid or liquid form of selumetinib is considered, the formulation in the presence of a scavenger radical, such as tocopherol, should reduce the degradation of selumetinib by photooxidation and/or oxidation. Furthermore, as we have been able to show that the degradation of selumetinib exposed to light is significantly reduced at acidic pH, it would also be relevant to take this parameter into account in pharmaceutical development.

## 4. Conclusions

We were asked to provide hospital formulations of selumetinib in liquid form to facilitate dosage adjustments and to consider, in a second phase, the development of topical forms for the local treatment of cutaneous symptoms of neurofibromatosis type 1. Thus, in addition to solubility aspects, another critical element to consider for feasibility was to evaluate the stability profile of the active substance in solution or suspension, to have prior knowledge that could effectively guide the pharmaceutical development.

This work, therefore, consisted of determining the behavior of selumetinib under stress conditions, conditions that were voluntarily more moderate than those found in the literature on this product, to seek to obtain a better predictive value of the data obtained. The results show that selumetinib in a liquid medium is particularly sensitive to oxidation and photooxidation, giving rise to the formation of two degradation products whose structure, fragmentation mechanisms by mass spectrometry and formation mechanisms in the presence of hydrogen peroxide and light irradiation were characterized. These data were then used as the basis for studies to stabilize selumetinib with the addition of compounds that could act as oxygen/radical scavengers and to determine the factors that effectively contribute to this, such as concentration and pH effects.

This work is only preliminary but should pave the way for further studies to achieve suitable formulations to ensure the quality of the formulations discussed.

## Data Availability

Not applicable.

## Supplementary Materials

The following supporting information can be downloaded at: https://www.mdpi.com/article/10.3390/pharmaceutics14122651/s1. Table S1: Tune data. Figure S1: LC-HRMS² mass spectrum of protonated selumetinib. Figure S2: LC-HRMS mass spectrum of protonated DP1. Figure S3: LC-HRMS^²^ mass spectrum of protonated DP1. Figure S4: LC-HRMS mass spectrum of protonated DP2. Figure S5: LC-HRMS² mass spectrum of protonated DP2. Figure S6: UV/visible spectra of selumetinib (final concentration = 6.25 µg·mL^−1^) at pH 3 (in red) and at pH 9 (in green).
