# Supplementary material for: Identification of the Major Degradation Pathways of Selumetinib
Source: Pharmaceutics. 2022 Nov 30;14(12):2651. doi: 10.3390/pharmaceutics14122651 (PMC9787286; doi:10.3390/pharmaceutics14122651)
Supplement: Supplementary file 1 [file pharmaceutics-14-02651-s001.zip › pharmaceutics-2045321-supplementary.pdf]

Supplementary Materials

Identification of the major degradation pathways of selumetinib

Tahar Sif eddine Bouchema [1], Maxime Annereau [1,2], Victoire Vieillard[3], Raphael Bouquet [3], Gisele Abreu Coelho [1], Florence Castelli [4], Audrey Solgadi [5], Muriel Paul [3,6], Najet Yagoubi [1], Philippe-Henri Secrétan[1,†,*], Bernard Do [1,3,†]

1 Université Paris-Saclay, Matériaux et santé, 91400, Orsay, France

2 Clinical Pharmacy Department, Gustave Roussy Cancer Campus, 114 rue Edouard Vaillant, 94800 Villejuif, France

3 Department of Pharmacy, Henri Mondor Hospital, AP-HP, Créteil, France

4 Département Médicaments et Technologies pour la Santé (DMTS), MetaboHUB, CEA, INRAE, Université Paris-Saclay, 91191 Gif-sur-Yvette, France

5 Université Paris-Saclay, Inserm, CNRS, Ingénierie et Plateformes au Service de l'Innovation Thérapeutique, 92296, Châtenay-Malabry, France.

6 EpidermE, Université Paris Est Creteil, 94010 Creteil, France.

Table of content

Structural elucidation of the main degradation products of selumetinib 3

Table S1: Tune data 4

Figure S1: LC-HRMS² mass spectrum of protonated selumetinib 4

Figure S2: LC-HRMS mass spectrum of protonated DP1 5

Figure S3: LC-HRMS² mass spectrum of protonated DP1 5

Figure S4: LC-HRMS mass spectrum of protonated DP2 6

Figure S5: LC-HRMS² mass spectrum of protonated DP2 6

Figure S6: UV/visible spectra of selumetinib (final concentration=6.25 µg.L^{-1}) at pH 3 (in red) and at pH 9 (in green) 7

Structural elucidation of the main degradation products of selumetinib

The structural elucidation of DPs was based on the comparison of neutral and/or radical losses to those experienced by selumetinib (Figure S1). The supporting spectral data are presented in Figures S2 to S5 .

Protonated DP1 and DP2 displayed accurate masses of 396.987 (Figure S2) and 441.997 (Figure S4), which could be assigned to $C_{17}H_{15}BrClFN_3O_3^+$ and $C_{15}H_{12}BrClFN_4O^+$, respectively. These results pointed that fluoride, bromide and chloride atoms were still present in both DPs, which was in favor of an absence of structural change on the cyclic parts and supported a degradation occuring only at the lateral chain.

LC-HRMS² experiments confirmed that the changes had only occurred on the lateral chain of selumetinib for both DP1 and DP2. Indeed, three ions shared the same accurate mass detected in the LC-HRMS² spectrum of selumetinib : 301.041, 344.991 and 379.960 (Figures S3 and S5), which pointed out that no change had happened on the cyclic parts of the drug. Conversely, for both degradation products, the base peak observed for selumetinib (exact mass: 361.009) and formed by loss of a part of the lateral chain was no longer detected in the LC-HRMS² mass spectra, confirming the changes had occurred in this part of selumetinib.

The difference between prototaned DP1 ($C_{17}H_{15}BrClFN_3O_3^+$) and the daughter ion 379.960 ($C_{15}H_9BrClFN_3O^+$) corresponds to a loss of ammonia undetected for selumetinib. Thus, this loss may easily be attributed to the formation of a primary amine function in DP1. Based on this result and on the fact that a change of structure should only happen in the lateral chain of selumetinib (see previous paragraph), DP1 was assigned as 5-((4-bromo-2-chlorophenyl)amino)-4-fluoro-1-methyl-1H-benzo[d]imidazole-6-carboxamide (Scheme 2).

The investigation of the LC-HRMS² mass spectrum (Figure S5) of protonated DP2 ($C_{15}H_{12}BrClFN_4O^+$) revealed three neutral loss unpreviously detected for selumetinib: a loss of oxirane, as highlighted by the daughter ion 397.970 ($C_{15}H_{11}BrClFN_3O_2^+$), folowed by either a neutral loss of water or a neutral loss of carbon dioxide (daughter ions of accurate mass 379.960, $C_{15}H_9BrClFN_3O^+$ and 353.980, $C_{14}H_{11}BrClFN_3^+$). The formation of the first daughter ion is explained by an internal rearrangement of the chain followed by a departure of oxirane. Following this withdrawal, the daughter ion can easily lose water or carbon dioxide. Based on these results, the structure of DP2 could be assigned as 2-hydroxyethyl 5-((4-bromo-2-chlorophenyl)amino)-4-fluoro-1-methyl-1H-benzo[d]imidazole-6-carboxylate (Scheme 2).

Table S1: Tune data

Spray Voltage (+):	3500,00
Spray Voltage (-):	3000,00
Capillary Temperature (+ or +-):	320,00
Capillary Temperature (-):	280,00
Sheath Gas (+ or +-):	50,00
Sheath Gas (-):	60,00
Aux Gas (+ or +-):	20,00
Aux Gas (-):	20,00
Spare Gas (+ or +-):	4,00
Spare Gas (-):	2,00
Max Spray Current (+):	100,00
Max Spray Current (-):	100,00
Probe Heater Temp. (+ or +-):	100,00
Probe Heater Temp. (-):	100,00
S-Lens RF Level:	50,00
Ion Source:	HESI

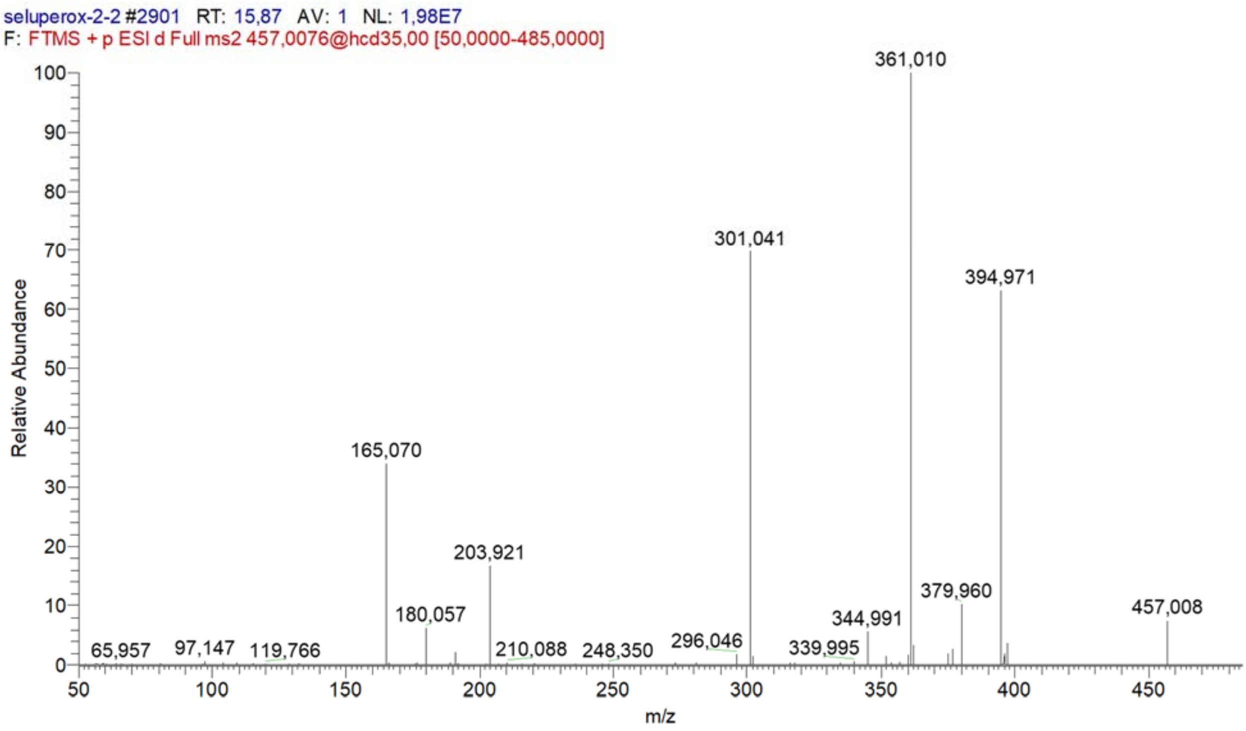

Figure S1: LC-HRMS^2 mass spectrum of protonated selumetinib

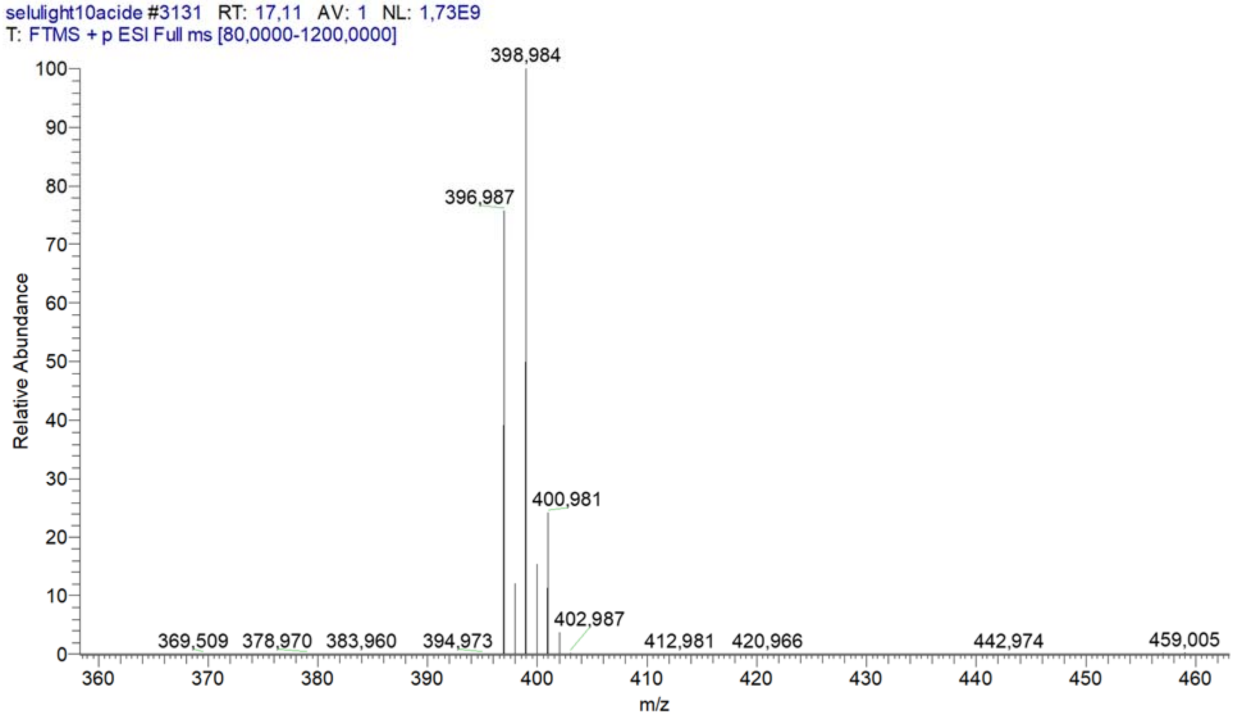

Figure S2: LC-HRMS mass spectrum of protonated DP1

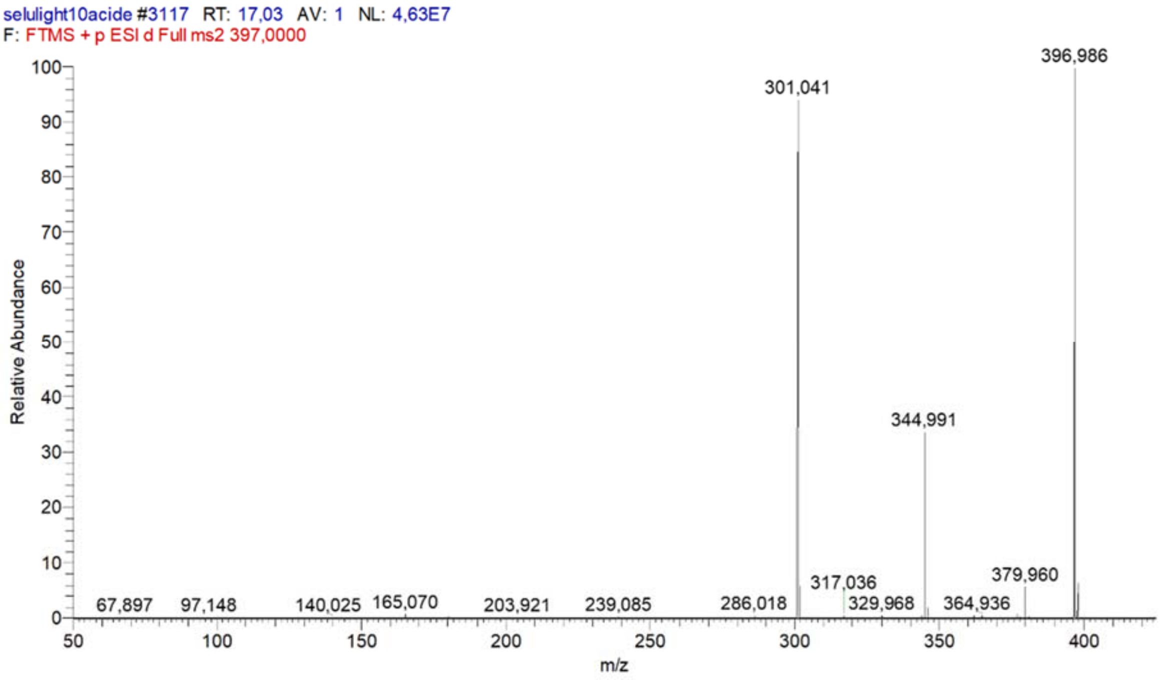

Figure S3: LC-HRMS^2 mass spectrum of protonated DP1

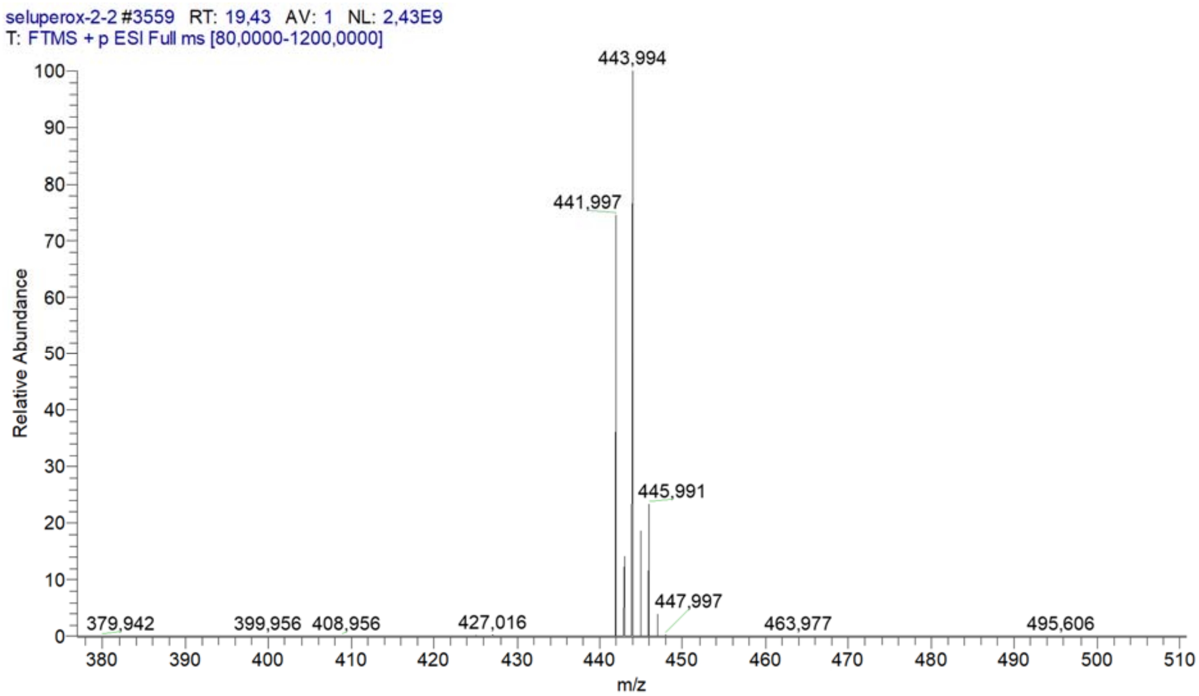

Figure S4: LC-HRMS mass spectrum of protonated DP2

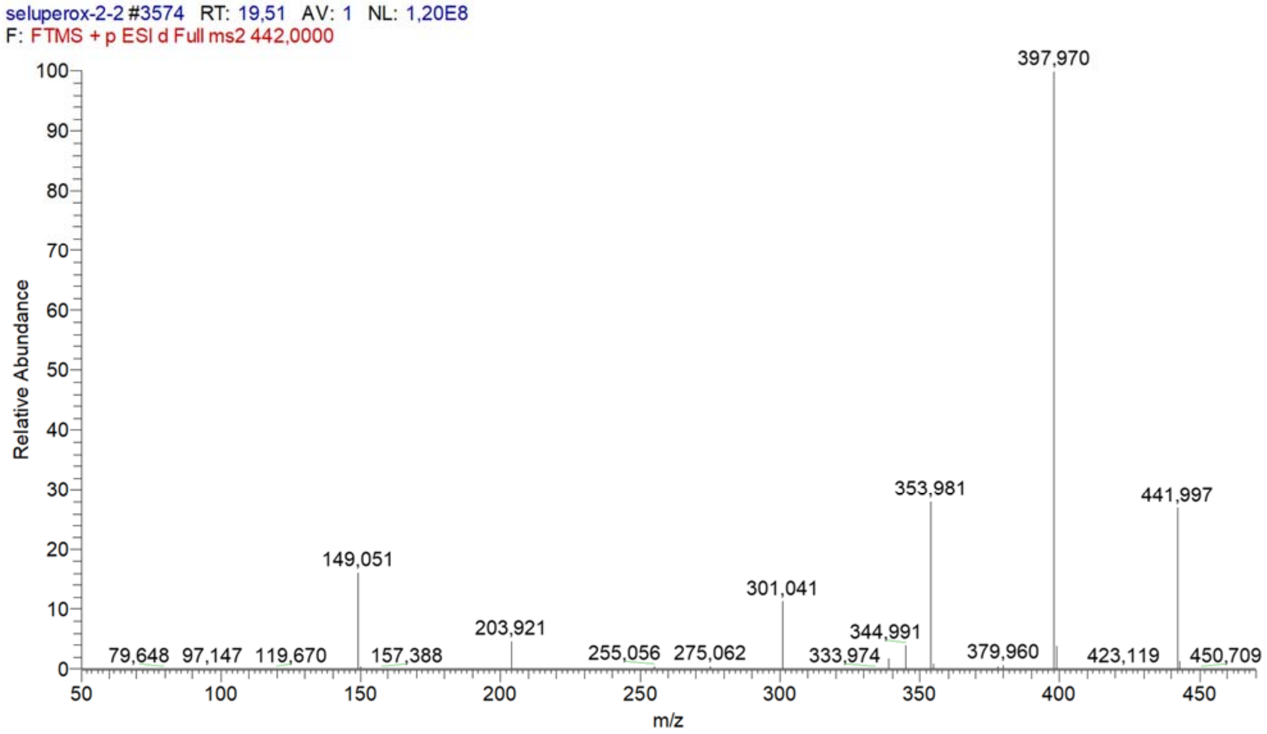

Figure S5: LC-HRMS^2 mass spectrum of protonated DP2

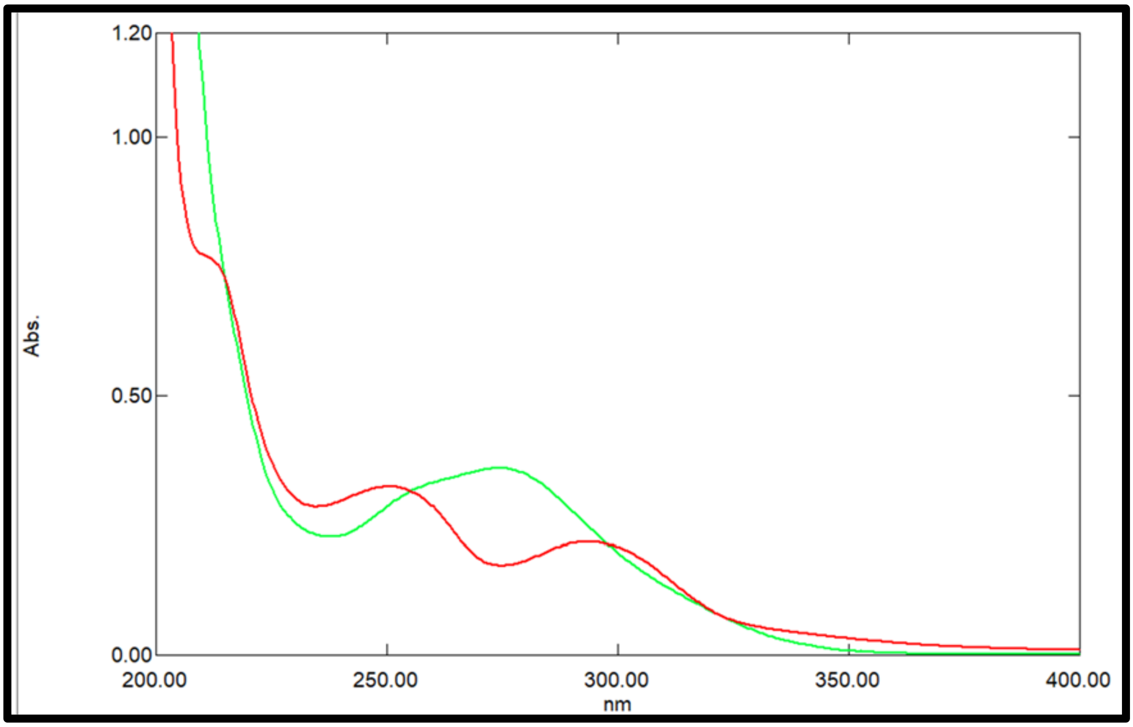

Figure S6: UV/visible spectra of selumetinib (final concentration=6.25 µg.mL^{-1}) at pH 3 (in red) and at pH 9 (in green)
